# Graph-Based Deep Learning Models for Predicting p*K*_a_ Values of Protein-Ionizable Residues via Physically Inspired Feature Engineering

**DOI:** 10.1021/acs.jcim.5c01681

**Published:** 2026-01-22

**Authors:** Ziyu Song, Ruixuan Wang, Xun Jiao, Zuyi Huang

**Affiliations:** † Department of Chemical and Biological Engineering, 8210 Villanova University, Villanova, Pennsylvania 19085, United States; ‡ Department of Electrical and Computer Engineering, Villanova University, Villanova, Pennsylvania 19085, United States

## Abstract

The p*K*_a_ value of a protein-ionizable residue reflects its potency to donate a proton at a given pH value, which is essential for understanding a wide range of biological activity. Therefore, the accurate prediction of p*K*_a_ values of protein residues is crucial for understanding enzymatic activity and protein–ligand binding, which are fundamental to drug discovery. Despite significant time and resources being invested to develop computational methods for protein residue p*K*_a_ prediction, the accuracy of existing tools, such as the widely used PROPKA, remains limited. In this study, an integrated framework that fuses molecular dynamics simulations and deep learning models is proposed to improve the predictive accuracy of p*K*_a_ values for ionizable residues. Specifically, we employ high-throughput molecular modeling using the AMOEBA polarized force field to construct a protein structure data set enriched with atomic electrostatics and other physics-inspired features. Using the experimentally determined p*K*_a_ values from the PKAD-2 data set, we trained three graph-based neural network models. All three models demonstrated substantial improvements in prediction accuracy across four ionizable residue types, aspartic acid, glutamic acid, lysine, and histidine, when compared to PROPKA3.5.1, with the graph attention networks-based model exhibiting both high accuracy and strong generalizability when benchmarking against several recently published machine learning models. Beyond these improvements in predictive performance, feature importance analysis of the best-performing models revealed physically meaningful patterns of the descriptive features that aligned with the underlying biophysical principles governing protein residue p*K*_a_ values, most notably, the complexity of the local microenvironment and the atomic geometric arrangement within the protein structure. Together, the trained p*K*_a_ models and the curated dipole moment-enhanced data set based on a polarizable FF offer a valuable resource for the research community, with potential applications in early-stage drug target identification and protein engineering.

## Graphical abstract

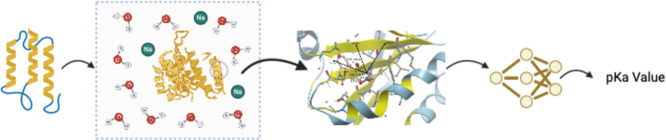

## 1 Introduction

The proteins are complex molecules that play many crucial roles in all biological organisms as they participate in almost every single physiological activity in living creatures. A broad spectrum of mechanisms mediating protein activities is regulated by their amino acid residues. For example, rhodopsin, a class G protein-coupled receptor (GPCR),^1^ is covalently bound to chromophore specifically at Lys296. Similarly, all rhodopsin analogues found in higher eukaryotes preserve a comparable active site around the lysine (Lys) residue.^2^ Additionally, certain cysteine (Cys) residues on human KEAP1 protein possess more potent cytoprotective functions than other Cys residues.^3,4^ Therefore, a deeper dive into the physicochemical properties of amino acids, such as the p*K*_a_ values of the amino acids with ionizable side chains, is urgently needed for understanding the mechanism of the associated proteins’ functions, given that amino acids with ionizable side chains constitute 30% of all protein residues.^5^ However, determining these p*K*_a_ values experimentally can be challenging, as it often requires specialized techniques such as nuclear magnetic resonance or potentiometric titrations. All these experimental techniques can be time-consuming and expensive and may not always be feasible for large or unstable proteins. As a result, extensive efforts and resources have been put into developing computational methods to predict the p*K*_a_ values of ionizable protein residues.

In general, computational approaches for predicting the p*K*_a_ of protein residues can be categorized into two main types. The first category is the empirical model, which relies on template p*K*_a_ values for each type of protein residue and estimates the p*K*_a_ shifts of the local environment effect, such as hydrogen bond formation and the desolvation effect, for the same type of residue being predicted. One notable example is PROPKA, developed by the Jensen group,^6–8^ which is a high-speed p*K*_a_ prediction method capable of predicting the p*K*_a_ values of all ionizable residues in a protein within seconds. Being more accurate than the first category, the second main category utilizes theoretical methods that predict p*K*_a_ values by estimating the free energy perturbation between protonated and deprotonated states of an ionizable residue (or titratable group) upon the transfer from an aqueous environment into the interior of a folded protein,^9^ through molecular mechanics (MM) modeling. These methods typically deployed conventional additive force fields (FFs) such as AMBER,^10^ GROMOS,^11^ and CHARMM.^12^ In this framework, electrostatic interactions between protein residues are characterized by predefined partial atomic charges assigned by the specific FF, enabling calculations of the desolvation and interaction energies that are critical for p*K*_a_ prediction. One of the most common theoretical simulation methods is constant-pH molecular dynamics (CpHMD) simulation.^13^ It enables dynamic sampling of the protonation states of ionizable residues so that the coupled effects between residue protonation and conformation dynamics were captured, allowing for more accurate estimation of p*K*_a_ values across different pH conditions.

While the molecular dynamics (MD)-based approaches for predicting protein residue p*K*_a_ values have demonstrated general effectiveness and reliability, the fixed partial charge representation employed in conventional FFs does not account for electrostatic variations induced by the surrounding environments that arise from interactions with neighboring atoms, transitions between aqueous and solid surrounding environments, and most critically, the effects of protein residue spatial rearrangements. These fixed-point charge models thus fall short in capturing the complex many-body interactions driven by atomic charge polarizations. As a result, they are not ideal for simulating electrostatically dominated phenomena,^14^ a category that includes the p*K*_a_ values of protein residues, i.e., the primary focus of this study. Instead of relying on conventional FFs used in classical MD simulations, certain studies have demonstrated that incorporating atomic multipole-based polarizations into the FFs can significantly improve the accuracy of p*K*_a_ predictions for protein residues.^15–17^ The polarizable FFs employed in these studies dynamically update atomic charges and dipole moments based on the local environment, allowing a more realistic simulation of electrostatic effect.^15,18^ Motivated by these studies, we employed the AMOEBABio18 polarized FFs,^19,20^ implemented in the Tinker 8.11.3 MD package,^21^ to perform high-throughput molecular simulation for physics-based protein residue data set generated in our work. This approach enables the calculation of induced dipole moments for each atom and some other features like atomic spatial positions during the molecular simulation process, thereby capturing dynamic atomic polarization and improving the representation of electrostatic interactions, a key factor in accurate p*K*_a_ prediction.

Building on the features extracted from AMOEBABio18-based MD simulations, we trained graph neural network (GNN) models using different architectures for protein residue p*K*_a_ prediction. With the recent rapid advancement of machine learning and deep learning, GNNs have been widely adopted in different fields, including but not limited to drug discovery,^22^ social networks^23^ and compound–protein interaction predictions.^24^ What is more, various GNN models have been proposed to address variant tasks such as node classification and graph classification. Among them, the graph convolutional network (GCN), which was first proposed by Kipf et al.,^25^ applies message passing schemes to aggregate information from neighboring nodes for node representation learning. The graph isomorphism network (GIN)^26^ combines GNNs and multi-layer perceptron (MLP) to enhance the expressive power in capturing complex graph structures. Moreover, graph attention networks (GAT), including two recently published studies for p*K*_a_ prediction,^27–29^ incorporate attention mechanisms^30^ to assign learnable weights to different neighbor nodes, enabling the models to perform more selective and efficient feature aggregation. To fully leverage these advanced graph-based models, we introduced a graph representation for protein residues by defining a universal local coordinate frame. This representation effectively reduces the internal noise in atomic coordinate data, enhancing the consistency and interpretability of structural features. Using this improved representation, we trained three GNN architectures—GCN, GIN and GAT on features extracted from AMOEBABio18-based MD simulations, along with experimentally determined p*K*_a_ values from the PKAD-2 database.^31^ The predictive performance of our models was benchmarked against widely used p*K*_a_ prediction tools like PROPKA, and the deep learning-based DeepKa.^32–34^ The models developed in this work, together with the physics-informed residue-level data set and the novel coordinate transformation framework, can pave the way for achieving more accurate and physically interpretable p*K*_a_ predictions that are particularly valuable for understanding protein function and accelerating early-stage drug discovery.

## 2 Materials and Methods

To accurately predict the p*K*_a_ values of ionizable residues in proteins, we developed an integrated computational pipeline (as shown in Figure 1) that combines physics-based molecular simulations with advanced GNNs. Starting from experimentally validated p*K*_a_ values in the PKAD-2 database, we retrieved corresponding protein structures from the RCSB protein data bank (PDB) and performed structural corrections using tools including UCSF ChimeraX,^35^ Swiss-PDBViewer,^36^ and PDBFixer.^37^ These curated structures were converted into Tinker-compatible formats and subjected to energy minimization using the AMOEBABio18 polarizable FF within the Tinker MD suite, enabling the capture of dynamic electrostatics and induced dipole moments critical for p*K*_a_ estimation. For each ionizable residue, a local spherical environment was extracted and transformed into a universal coordinate frame to eliminate the structural noise. These environments were encoded as graphs, where atoms serve as nodes enriched with 26 physicochemical features—including one-hot encoded residue type, coordinates, dipole vectors, and hydrogen bonding profiles—while edges represent chemical bonds. The resulting graph representations were used to train three distinct GNN architectures (GCN, GIN, and GAT), each optimized via a grid search and evaluated using cross-validation. The details of individual steps in this pipeline are provided in the subsections below.

**Figure 1:**
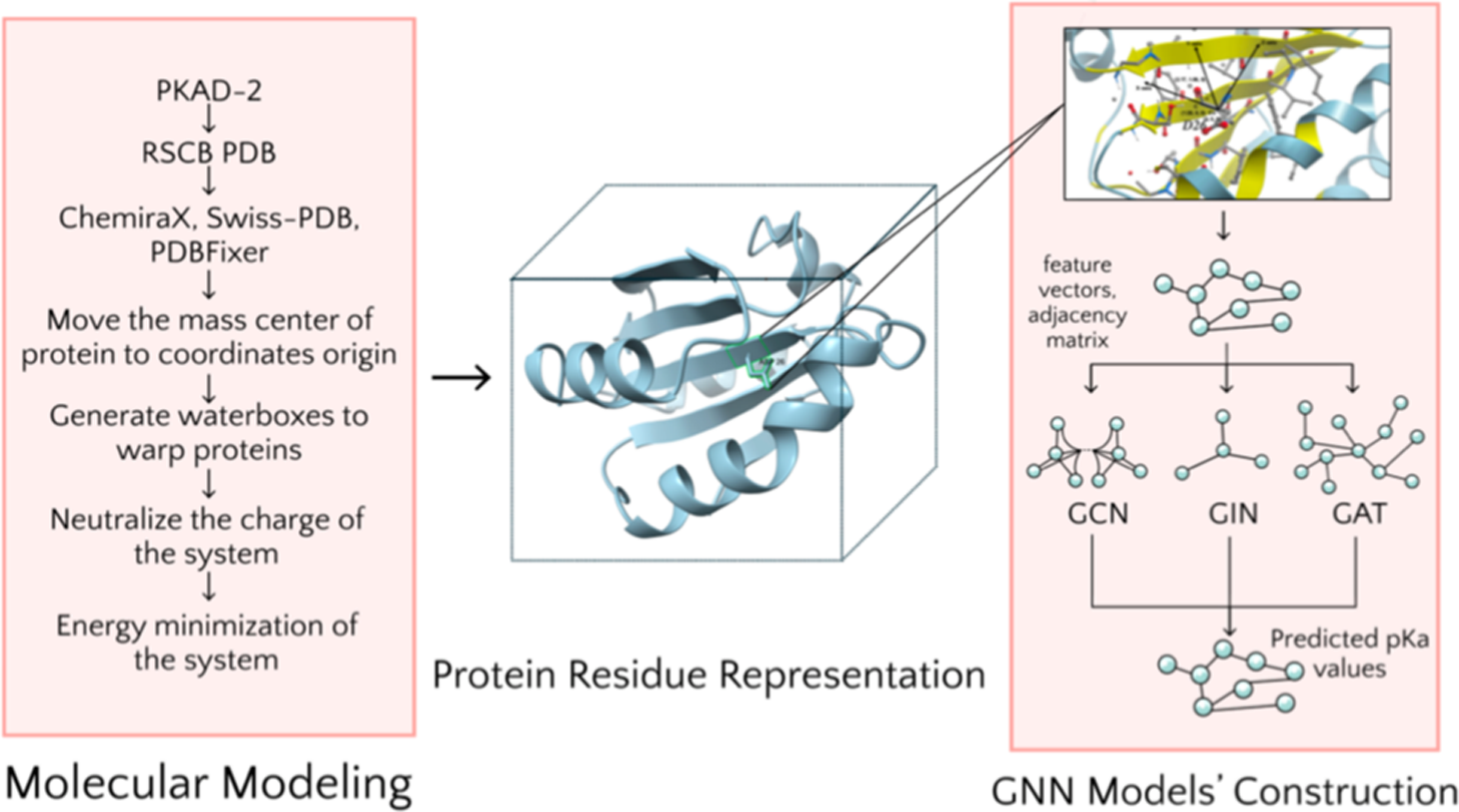
Overview of the computational pipeline. The process begins with the extraction of PDB IDs from the PKAD-2 database, followed by PDB file correction and preprocessing for high-throughput MD simulations using Tinker 8.11. Local environments are then constructed for each residue to generate graph-based representations of protein residues. Finally, graph neural network models based on three architectures—GCN, GIN, and GAT—are trained on these representations to predict p*K*_a_ values for protein residues.

### 2.1 Data Set Construction

This study utilized the PKAD-2 database^31^ that contains 1378 experimentally determined p*K*_a_ values of ionizable residues from 165 wild-type proteins, along with additional 269 p*K*_a_ values from mutated proteins. The PDB IDs^38^ of the wild-type proteins in the data set were obtained to extract the experimentally determined protein constructs. During the data preprocessing and molecular simulations, certain proteins and their corresponding amino acid residues were excluded from further analysis. For example, structures with PDB IDs 1IGC, 1HNG, 1YPI, and 1BHC were removed from the data set due to non-convergent dipole moment calculations, which resulted from problematic PDB structures containing atoms positioned too closely together. Additionally, cysteine and tyrosine residues were excluded due to their limited occurrences in the PKAD-2 database (20 and 45 entries, respectively), which were insufficient for reliable model training. Although excluded from the present analysis, we acknowledge their substantial biochemical importance, particularly the critical role of cysteine in drug discovery, where it is frequently targeted in covalent inhibitor design.^39^ Residues corresponding to C– and N– terminus were also filtered out to ensure consistency across the data set. The final curated data set used for training and evaluation comprised 1167 residues across four ionizable residue types, aspartic acid (Asp), lysine (Lys), glutamic acid (Glu), and histidine (His), with their p*K*_a_ value distributions summarized in Table 1.

**Table 1:** Distribution of Samples across the Four Residue Types^a^

residue	samples	p*K*_a_ values range	mean p*K*_a_ value
Asp	382	[0.5, 9.9]	3.43
Lys	148	[6.5, 12.12]	10.69
Glu	397	[2.1, 7.2]	4.12
His	240	[2.3, 9.19]	6.42

a For each residue type, the mean and p*K*a value range are also reported.

### 2.2 Protein Structures’ Preprocessing for Molecular Simulation

The structures of all proteins containing the four residue types (Asp, Lys, Glu, and His) with experimentally determined p*K*_a_ values recorded in the PKAD-2 database were retrieved from PDB and subjected to preprocessing for subsequent molecular simulation. The PDB files were initially processed using PDBFixer, a molecular structure preparation tool included in the OpenMM7 python toolkit,^40^ to identify and add missing heavy atoms and residues, as well as to replace non-standard residues with their corresponding canonical amino acids. Subsequently, the structures were further cleaned using ChimeraX,^40^ during which water molecules were removed while essential ions such as Cu and Fe— necessary for preserving metal bonds—were retained. Some PDB files encountered errors or produced anomalous structures during standard preprocessing. For instance, the structure of PDB 1I0E displayed an abnormally extended terminal region after processing with ChimeraX. Such problematic cases were manually corrected using SWISS—PDB Viewer.^41^

### 2.3 Dipole Moment Calculation via Molecular Modeling

#### 2.3.1 Tinker Molecular Modeling and AMOEBA Polarizable Force Field

The MD simulations in this study were conducted using Tinker 8.11, a comprehensive molecular modeling package designed to perform a wide range of classical MM calculations and MD simulations. It supports both widely used FFs, such as those from AMBER, CHARMM, and GROMOS, as well as advanced polarizable FFs, including the series of polarizable atomic multipole AMOEBA FFs and the more recent AMOEBA + FF,^41^ which incorporates charge penetration effects. The specific FF employed in this study, AMOEBABio18, has the potential energy formula described in detail by,^20,42^ as presented in eq 1

1 $$U=U_{bond}+U_{angle}+U_{b-a}+U_{oop}+U_{torsion}+U_{vdW}+U_{ele}^{perm}+U_{ele}^{ind}$$

where the valence bonding contributions are composed of five terms, bond stretching *U*_bond_, angle bending *U*_angle_, bond-angle cross coupling *U*_b–a_, out-of-plane distortions *U*_oop_, and torsional rotations *U*_torsion_. The pairwise additive van der Waals *U*_vdW_ are modeled using the buffered 14–7 function,^43^ which offers a smoother repulsive phase compared to the standard Lennard–Jones 12–6 form. Notably, instead of relying on single point Coulombic electrostatics deployed in conventional FFs, the *U*_ele_ in AMOEBA FF is split into two parts: *U*_ele_^perm^ and *U*_ele_^ind^. The permanent electrostatics, *U*_ele_^perm^, are computed through multipole–multipole interactions, where each atom carries not just a partial charge (monopole) but also an associated dipole vector and quadrupole tensor, capturing a more detailed electrostatic spatial representation of the molecular system. What is more, each atom is assigned a distinct isotropic polarizability, which determines its induced atomic dipole moments *U*_ele_^ind^, in response to the local electrostatic environment.^20^ Following the previous preprocessing steps, the resulting PDB files were converted to Tinker compatible topology files (.xyz) using the AMOEBABio18 parameter set.^20,42^ Subsequently, a cubic water-box was either selected from a library of pre-equilibrated AMOEBA explicit water-boxes of various sizes or generated and equilibrated specifically for oversized systems, ensuring that each protein structure was fully encapsulated with at least 1 nm of solvent padding on all sides. The proteins in topology files were then soaked into the corresponding water-boxes according to their sizes, and the entire systems were neutralized to achieve a net charge balance.

#### 2.3.2 System Energy Minimization and Dipole Moments’ Generation via Tinker

After the PDB files of all proteins from the PKAD-2 database have been processed as described above, each protein system underwent an EM process, using a convergence root mean square (RMSE) gradient threshold set to 1.0 kcal/mol/Å according to Tinker’s user guide. During the EM step, the atomic coordinates were iteratively adjusted to eliminate unfavorable steric clashes and high-energy configurations that are physically unrealistic, thereby yielding a stable, energy-minimized structure for each protein for subsequent data set construction. Additionally, the induced atomic dipole Moments μ_*i*,α_^ind^ were calculated during the EM process using eq 2

2 $$\mu_{i,\alpha }^{ind}=\alpha_{i}\left(\sum_{j}T_{\alpha }^{ij}M_{j}+\sum_{j}T_{\alpha \beta }^{ij}\mu_{j,\beta }^{ind}\right)$$

where α_*i*_ is the isotropic polarizability of atom *i* in the α direction. The local electric field at site *i* is derived from contributions of both the permanent electrostatic (*M*_*j*_) of surrounding atom *j* and the induced electrostatic μ_*j*,β_^ind^(the dipole moments caused by neighboring atom *j* on the β direction), via the interaction tensors *T*_α_^*ij*^ and *T*_αβ_^*ij*^ between atom *j* and atom *i*, respectively. The resulting induced dipole moment vector μ_*i*,α_^ind^ is expressed in SI units of Coulomb-meter (C·m). The Debye (*D*) is also a commonly used unit, with 1*D* equal to 3.33564 × 10^–30^ C·m.

The sizes and potential energies of all the proteins following EM are summarized in Figure 2. The system sizes range from 45 Å to 196 Å per axis. An overall decreasing trend in potential energy is observed with increasing system size. The magnitudes of potential energies fall in the range 10^5^ to 10^6^ kJ·mol^–1^ and are consistently negative, reflecting the stability and validity of the energy minimized structures.

**Figure 2:**
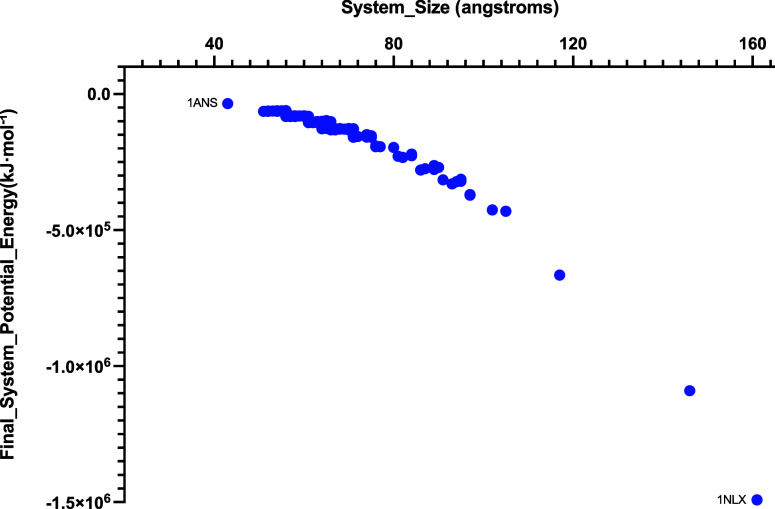
Summary of the final system potential energy and sizes for all proteins; the smallest protein, 1ANS, was solvated in a 45 Å × 45 Å × 45 Å Tinker pre-equilibrated water-box, resulting in a final potential energy of −34,754 kJ·mol^–^1. The largest system 1NLX was encapsulated in a 161 Å × 161 Å × 161 Å self-generated and equilibrated water-box, with a corresponding final potential energy of −1,491,257.6 kJ·mol^–^1.

### 2.4 Local Environment Representations of Residues Using a Novel Local Coordinate Frame

After the EM processes of all proteins, spherical regions centered on the C_α_ atom of the target residue were cropped from each corresponding protein to construct the local environment of the interested ionizable residues. To optimize this spherical representation, five feature data sets were generated by cropping spheres with the radii of 7 Å, 8 Å, 9 Å, 10 Å, and 11 Å. The extracted feature data sets were subsequently used to evaluate the impact of spherical radius on p*K*_a_ prediction through deep learning models.

To establish a consistent and noise-reducing spatial representation of each ionizable residue, a “*X*–*Z*–*Y*” local coordinate frame was defined for every cropped residue sphere (Figure 3). This transformation leverages the planarity of the peptide bond and applies the right-hand rule to create a universal reference system. The partial double-bond nature of the peptide bond restricts rotation, causing six key atoms, including the C_α_ and carbonyl group (–CO–) of residue *i*, along with the amide nitrogen (–NH–) and C_α_ of residue *i* + *1*, to lie approximately in the same plane. Based on this geometric constraint, the local coordinate frame is constructed as follows: (1) the origin is placed at the C_α_ atom of the target residue; (2) the *X*-axis is defined as the normalized vector from the C_α_ to the carbonyl carbon of the same residue; (3) the *Z*-axis is the unit vector perpendicular to the plane formed by the *X*-axis and the carbonyl group; and (4) the *Y*-axis is calculated as the cross product of the *X*- and *Z*-axes to ensure an orthonormal right-handed coordinate system. This reference frame establishes a consistent geometric context for downstream feature extraction and graph representation that is invariant to protein rotation, with all C_α_ atoms positioned at the origin (0, 0, 0), and the carbonyl C and O atoms placed at approximately (1.53, 0, 0) and (2.17, 1.06, 0) respectively, maintaining a relatively fixed geometry.

**Figure 3:**
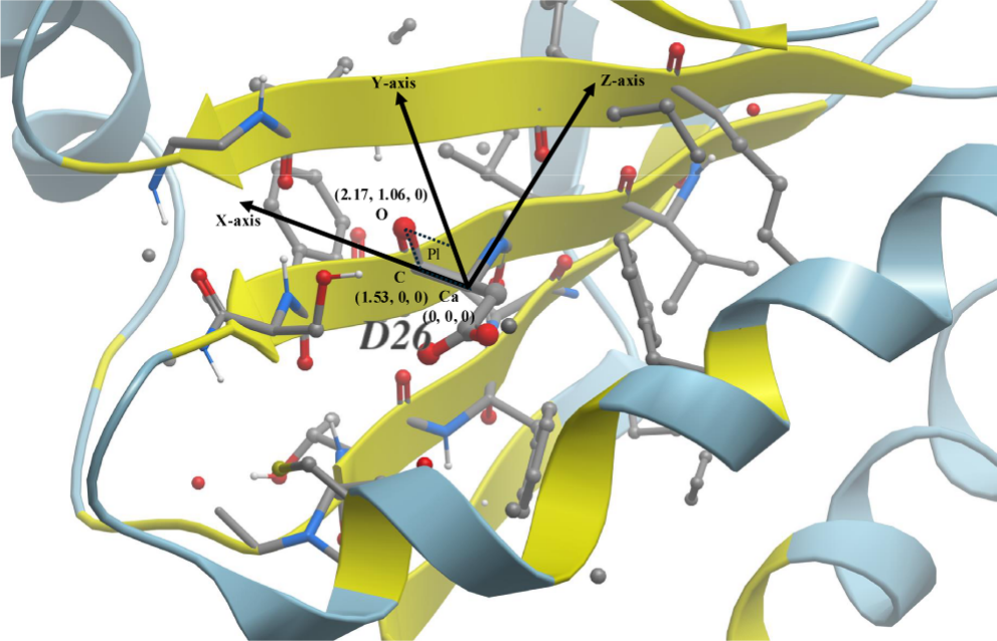
Illustration of the universal local coordinate frame using Asp26 from the protein 1ERT as an example. All yellow-colored residues lie partially or entirely within a 9 Å radius to the C_α_ of Asp26, which serves as origin of the coordinate frame and defines the local environment. Residues in blue fall outside this spherical region and are excluded from the local environment in this case.

All of the constituent atoms of each residue were subsequently transformed into their respective local coordinate frames. The *X*–*Y*–*Z* coordinates of each atom were re-calculated relative to the C_α_ atom and the defined local axes. This transformation captured the intrinsic geometric arrangement of atoms within each residue in a consistent and system-independent manner across different proteins, enabling a uniform representation of residues across different protein structures and effectively eliminating internal noise in the coordinates data; i.e., the atoms with identical positions relative to the C_a_ atom could have totally different coordinates from different proteins.

### 2.5 Graph-Based Data Representation of Protein Residues

The DL models have demonstrated great success in predicting molecular properties such as protein–ligand binding affinity^44^ and p*K*_a_ values.^32–34^ Recently, graph-based deep learning models are widely applied in the drug delivery and protein analysis field.^28,29,45^ Building on these successes, this study proposes the use of graph-based deep learning models for effective p*K*_a_ prediction. Different from the existing studies, we focus on individual protein residues, rather than the entire protein as a whole as done in AlphaFold.^45^ In our approach, each protein residue is represented as a graph

3 G={V,E}

where *G* is the protein residue, *V* denotes the atoms of the target residues, and *E* represents the chemical bonds formed between them (edges). To numerically represent the previously processed residue local environments, each cropped residue sphere was further represented in this graph-based format. This representation retains the intrinsic topological structure of each protein residue while allowing flexible and efficient integration into graph-based DL pipelines.

For each node (atom) in the graph representation of the target residue, a set of physically informed features were extracted from the cropped local environments of the residues after the above EM process. These features include the induced dipole moment vectors and the normalized atomic coordinates along the *X*-, *Y*-, and *Z* axes. The MDAnalysis toolkit^46,47^ was used to extract the following features: the number of heavy atoms (C, N, O, and S) from neighboring residues located within the certain radii of the corresponding atom and solvent accessible surface area (SASA) of each atom on the corresponding target residue. In addition, the program MDTraj^48^ was utilized to quantify hydrogen bonding features at the atomic level. For each atom, hydrogen bonds were counted separately based on its role as a hydrogen donor or acceptor during hydrogen bond formation, reflecting the atom’s distinct electronegativity characteristics. In total, the following 26 node-level features were extracted to represent the structural and physicochemical properties of each target residue: four binary variables to account for the four possible amino acid residue types (Features 1–4); the *X*, *Y* and *Z* coordinates of the atom after local-frame transformation (Features 5–7); *X*, *Y*, and *Z* coordinates of the induced dipole moment vectors of the atom (Features 8–10); the number of heavy atoms (C, N, O, and S) from neighboring residues within the five radii of each atom, i.e., 7 Å, 8 Å, 9 Å, 10 Å, and 11 Å (Features 11–14 for the five radii); the number of hydrogen bonds formed on each atom, either as a hydrogen acceptor or a donor (Features 15–16); the SASA of each atom (Feature 17); and one-hot encoding of atom types for N, C, O, H, S, with the same type of atom (N, C, O and H) differentiated by location-backbone vs side chain (Feature 18–26). The p*K*_a_ value of the corresponding residue served as the graph-level label. Features 11 to 16 were normalized across the entire data set immediately after extraction, while the others were not normalized.

In addition to the aforementioned node-level features, the adjacency matrix is essential in capturing interconnectivity between nodes (atoms) within each graph and aggregating information from neighboring nodes (atoms). An adjacency matrix is a square matrix of shape *N* × *N*, where *N* denotes the index of the atoms in the graph. For example, the residue Gln20 from protein 1ANS has an adjacency matrix shown in Figure 4 where 1 indicates the two atoms are chemically bonded and 0 means no chemical bond is formed between them.

**Figure 4:**
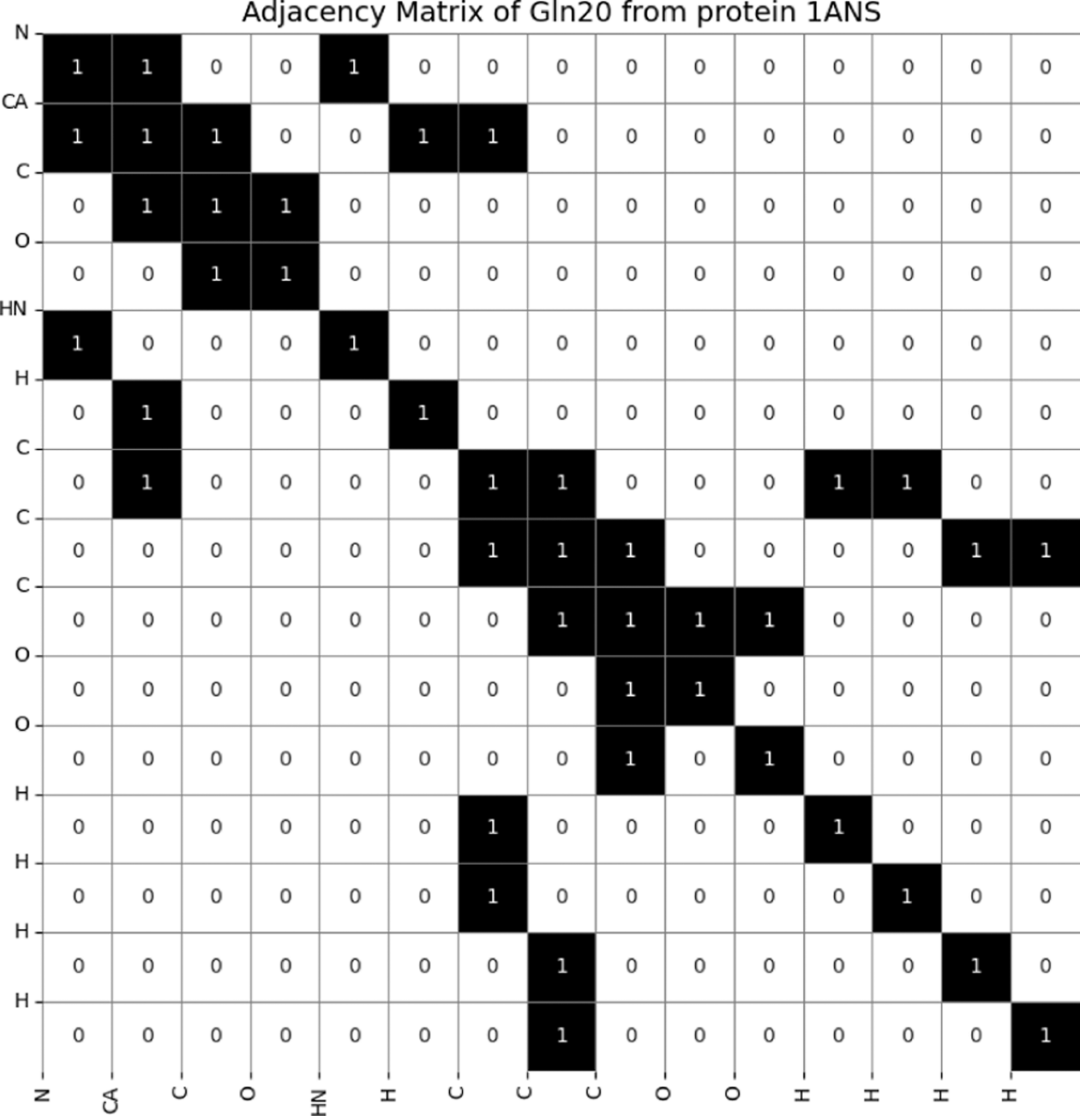
Adjacency matrix for GNN using Gln20 from protein 1ANS as an example. Each 1 indicates the presence of a chemical bond between two atoms, while 0 indicates no bond. It is worth noting that the 1s along the diagonal of the adjacency matrix represent self-loops, i.e., edges connecting each node (atom) to itself. However, they are not physically present in the molecule but are intentionally included here to allow the node (atom) to retain its own information during the process of feature aggregation from neighboring nodes during model training.

### 2.6 Graph-Based Deep Learning Model Constructions and Hyperparameters’ Tuning

In this paper, we utilized GNN-based approaches for protein p*K*_a_ prediction. In particular, we utilized three graph learning models, GCN, GIN, and GAT, with different architectures and feature aggregation mechanisms and established comprehensive grid search experiments to achieve better performance through hyperparameter tuning of GNN architectures. All GNN models are implemented with three layers: an input layer, a single hidden layer, and a final output layer. Batch training was performed using the Adam optimizer.^49^ However, batch normalization was intentionally omitted, as several features, such as the *X*, *Y*, and *Z* coordinates and the dipole moment vectors of atoms, were designed to retain their absolute values to capture the critical structural and electrostatic properties of the residues. Additionally, some features had already been prenormalized, as described earlier, making further normalization unnecessary. Moreover, to prevent potential overfitting in model constructions, we employed 10-fold cross-validation (CV) and early stopping (with a patience of 20) during the model training, in addition to applying dropout in the hidden layer. Each model was trained using the same set of nodes (atom) feature vectors described above, and a comprehensive grid search was performed to optimize the hyperparameters for each model.

The GCN models update node features according to eq 4

4 $$h_{i}^{\left(l+1\right)}=\sigma \left(\sum_{j\in N\left(i\right)\cup \left\{i\right\}}\frac{1}{\sqrt{\left|N\left(i\right)\right|\left|N\left(j\right)\right|}}W^{\left(l\right)}h_{j}^{\left(l\right)}\right)$$

where *h*_*i*_^(*l*+1)^ denotes the updated feature vector of node *i* at layer *l* + 1 and σ corresponds to the rectified linear unit (ReLU) activation function to introduce non-linearity in this study. The summation aggregate messages from all first-order neighboring nodes j∈N(i) and node *i* itself (with self-loop), transformed by a shared learnable weight matrix *W* ^(*l*)^, and scaled by the normalization factor $\frac{1}{\sqrt{\left|N\left(i\right)\right|\left|N\left(j\right)\right|}}$ , which accounts for the degrees (i.e., number of neighbors) of node *i* and *j*.

During the hyperparameter tuning process, all GCN models shared the same architecture: starting with an input layer with the dimension of input data, followed by two graph convolutional layers. The first GCN layer was set with a tunable number of hidden channels, followed by a ReLU activation to introduce nonlinearity and a dropout layer for regularization. The second GCN layer outputs the predictions at the node level, which are subsequently aggregated using global mean pooling to produce the final graph level prediction of the residue’s p*K*_a_ value.

Meanwhile, unlike GCN models, which use a simple weight matrix to aggregate information from neighboring nodes, GIN models aggregate information via an MLP Layer. The node feature update rule for GIN is defined in eq 5

5 $$h_{i}^{\left(l+1\right)}=MLP^{\left(l\right)}\left(\left(1+\varepsilon^{\left(l\right)}\right)\cdot h_{i}^{\left(l\right)}+\sum_{j\in N\left(i\right)}h_{j}^{\left(l\right)}\right)$$

where *h*_*i*_^(*l*+1)^ denotes the feature vector of node *i* at layer *l* + 1 and ε^(*l*)^ is a learnable scalar that scales the contribution of the central node’s own features. The sum aggregates feature vectors from all neighboring nodes j∈N(i) , and the combined result is transformed through a layer-specific MLP^(l)^. In this study, the output size of the MLP was set as a tunable parameter, followed by a RELU activation function and a dropout layer for regularization. After the graph convolutional layer, a global pooling function was applied and a fully connected (FC) layer was used to output the final p*K*_a_ prediction. Different from the grid search for the GCN models mentioned above, the global pooling function was further considered as a tunable parameter.

Different from the GCN and GIN approaches, the GAT model replaces the static weights determination with dynamic weights computed from node features through an attention mechanism.^30^ To stabilize the learning process and to enhance the model’s ability to capture complex relationships, multihead attention is employed in this study. In particular, the node feature update process in the GATv2Conv model^50^ is more expressive than the original GAT. The feature update for node *i* is given in eq 6

6 $$h_{i}^{\prime }=|{|}_{m=1}^{M}\left(\alpha_{i,i}^{\left(m\right)}W_{s}^{\left(m\right)}h_{i}+\sum_{j\in N\left(i\right)}\alpha_{i,j}^{\left(m\right)}W_{t}^{\left(m\right)}h_{j}\right)$$

where *h*_*i*_^′^ is the updated feature of node *i* and ||_*m*=1_^*M*^ indicates the concatenation across heads *m*∈{1,···,*M*}. For each head, α_*i*,*i*_^(*m*)^ is the attention coefficient for the self-loop, α_*i*,*j*_^(*m*)^ is the attention coefficient from node *i* (query node) to the neighbor node *j* (key node), and *W*_*s*_^(*m*)^ and *W*_*t*_^(*m*)^ are the shared learnable weight matrix that transforms the features of the source node *i*’s own features and target neighbor nodes, respectively. The attention coefficient α for each head *m* is calculated using a Softmax-like probability distribution, as shown in eq 7

7 $$\alpha_{i,j}^{\left(m\right)}=\frac{\exp\left({\left(a^{\left(m\right)}\right)}^{T}\cdot LeakyReLU\left(W_{s}^{\left(m\right)}h_{i}+W_{t}^{\left(m\right)}h_{j}\right)\right)}{\sum_{j\in N\left(i\right)\cup \left\{i\right\}}\exp\left({\left(a^{\left(m\right)}\right)}^{T}\cdot LeakyReLU\left(W_{s}^{\left(m\right)}h_{i}+W_{t}^{\left(m\right)}h_{j}\right)\right)}$$

where ${\left(a^{\left(m\right)}\right)}^{T}$ is a learnable attention score vector for each head and LeakyReLU is the activation function (with a slope 0.2 for negative values) introducing non-linearity. In this case, the number of attention heads *M* was considered as a tunable hyperparameter as it influenced how the node features are distributed and how attention is computed for different subsets of the feature vectors.

In summary, our grid search for hyperparameter tuning of the GNN models explored a range of configurations to optimize the performance. The number of channels in the hidden layer was varied across 16, 32, 48, and 64, while batch sizes for training and evaluation were set to 16, 24, 32, and 40. Learning rates tested included 0.001, 0.006, and 0.1. Dropout rates in the hidden layer ranged from 0.2 to 0.5 to prevent overfitting. For the GIN models, both global mean pooling and global add pooling strategies were evaluated, and for the GAT models, the number of attention heads was treated as a tunable parameter with values of 4, 6, and 8. Additionally, three different loss functions from PyTorch^51^ were tested to guide model training: SmoothL1Loss (β = 0.5), L1Loss, and MSELoss. All the models generated during the tuning process were saved, and the models with the predictions for all of the samples from the validation batches with the lowest mean absolute error (MAE) and the RMSE were collected as the evaluation metrics. The models were trained on both the CPU (AMD Ryzen Threadripper 7970X) and GPU (Nvidia GeForce RTX 3080Ti).

## 3 Results and Discussions

The results section presents a comprehensive evaluation of three graph-based deep learning models—GCN, GIN, and GAT—trained to predict the p*K*_a_ values of ionizable residues using features derived from local residue environments. Through an extensive hyperparameter grid search and 10-fold cross-validation, the best-performing configurations for each model architecture were identified. These top models were then benchmarked against established p*K*_a_ prediction tools PROPKA and DeepKa. Feature importance analysis was further conducted using GNN Explainer,^52^ a program successfully applied to molecular property prediction task,^53^ to reveal physically meaningful features such as local atomic composition, spatial coordinates, and dipole moments that were hinted by the developed models. These results not only validate the accuracy and robustness of the proposed GNN-based framework but also highlight its interpretability in capturing the biophysical mechanisms underlying p*K*_a_ variation.

### 3.1 GCN Model Hyperparameters Grid Search Results

The 10-fold cross-validated results were collected from all the GCN models evaluated during hyperparameter grid tuning, and the distributions of MAE and RMSE values are presented in Figure 5, with the top end of the bar corresponding to the hyperparameter combination that yielded the highest MAE or RMSE among all the models trained on that data set, while the bottom end corresponds to the model with the lowest MAE or RMSE values. The line within the box indicates the median MAE or RMSE value. Among the evaluated GCN models, the best-performing configuration, referred to as GCN_1, was trained on Data set 2 with a sphere radius of 9 Å, achieving a MAE of 0.549 and an RMSE of 0.798. This optimal model utilized 64 hidden layer channels, a batch size of 16, a learning rate of 0.006, a dropout rate of 0.3, and MSELoss as the training objective function.

**Figure 5:**
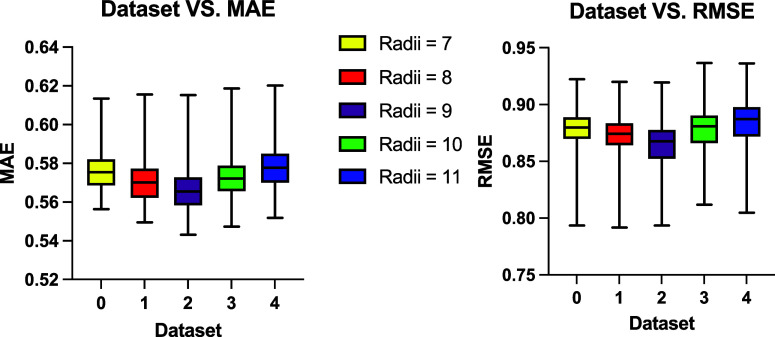
MAE and RMSE values from all GCN models trained across five data sets extracted and curated from PKAD-2 with five different spherical radii. The models yielded comparable performance across five data sets, with median MAE values between 0.57 and 0.58, and median RMSE around 0.87. The best-performing model was trained on Data set 2 (marked in purple with the radius of 9 Å), which also produced the overall best results, exhibiting both the lowest median MAE and median RMSE among all data sets.

### 3.2 GIN Model Implementation and Hyperparameters Grid Search Results

The same procedure described above was used to collect and analyze GIN hyperparameter grid tuning results, the results are summarized in Figure 6. The best GIN model was still trained using Data set 2, named GIN_1, achieving an MAE of 0.547 and RMSE of 0.786. This best-performing model was obtained using the following set of hyperparameters: it was trained with 64 hidden layer channels, a batch size of 16, a learning rate of 0.01, and a dropout rate of 0.5. The training objective function used was MSELoss, and the global pooling function applied was the global mean pool.

**Figure 6:**
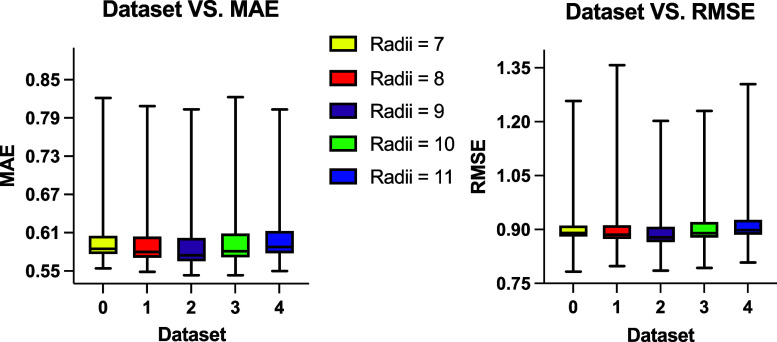
MAE and RMSE values from all GIN models trained across five data sets. The models yielded comparable performance across five data sets, with median MAE values between 0.57 and 0.59 that are similar to those from GCN models training and median RMSE around 0.9. The best-performing model was trained on Data set 2, which also produced the overall best results, exhibiting both the lowest median MAE and median RMSE across all data sets.

### 3.3 GAT Model Implementation and Hyperparameters Grid Search Results

A similar analysis to that described in Sections 3.1 and 3.2 was conducted for all GAT models evaluated during the hyperparameter tuning process, with the results shown in Figure 7. The best performing model, named GAT_1, achieved an MAE of 0.501 and RMSE of 0.716. This model was trained on Data set 3 using 48 hidden layer channels, a batch size of 32, a learning rate of 0.01, and a dropout rate of 0.5. The training objective function was MSELoss, and the model employed 4 attention heads in its architecture. The grid search results for all the hyperparameter combinations across three model architectures were collected and are provided in the appendix.

**Figure 7:**
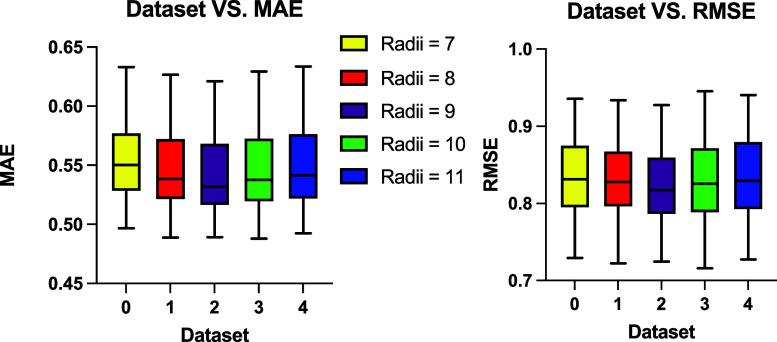
MAE and RMSE values from all GAT models trained across five data sets. The models yielded comparable performance across five data sets, with median MAE values between 0.52 and 0.55, and median RMSE around 0.82. The best-performing model was trained on Data set 3 with the lowest average MAE and RMSE, with models trained with Data set 2 yielding both the lowest median MAE and median RMSE.

Overall, the GAT models exhibited the best performance, achieving both the lowest and median MAE and RMSE values. The GCN and GIN showed similar overall results. Among the three architectures, the GCN and GAT models produced more stable outcomes, with MAE and RMSE values varying within a narrow range of 0.15 units. In contrast, several GIN models exhibited significant deviations from the others, resulting in a wider range of MAE and RMSE values of approximately 0.4 units. The grid search results for all three architecture models are provided in the Supporting Information (Figures S1–S3), along with the training/evaluation loss histories across epochs for GAT_1 (Figure S4).

### 3.4 Benchmarking GNN-Based Models against Established p*K*_a_ Predictors

The predicted p*K*_a_ values for the entire data set were obtained during the GAT_1 model training process. To further assess the model performance, these predictions were compared with the corresponding values generated by PROPKA 3.5.1 for each residue type (Figure 8). The GAT_1 model exhibited substantial improvements in MAE for all residues, particularly for Glu and His, where errors decreased by more than 36% compared to PROPKA predictions. Asp and Lys also showed significant improvements, with MAE reductions of approximately 23% and 29%, respectively. Similarly, from the RMSE perspective, the GAT_1 model also outperformed PROPKA across all residue types, especially for Glu and His, with RMSE reductions at approximately 44% and 31%, respectively. For Asp and Lys, the GAT_1 model achieved more modest yet meaningful gains, with RMSE improvements of around 21% and 13%, respectively.

**Figure 8:**
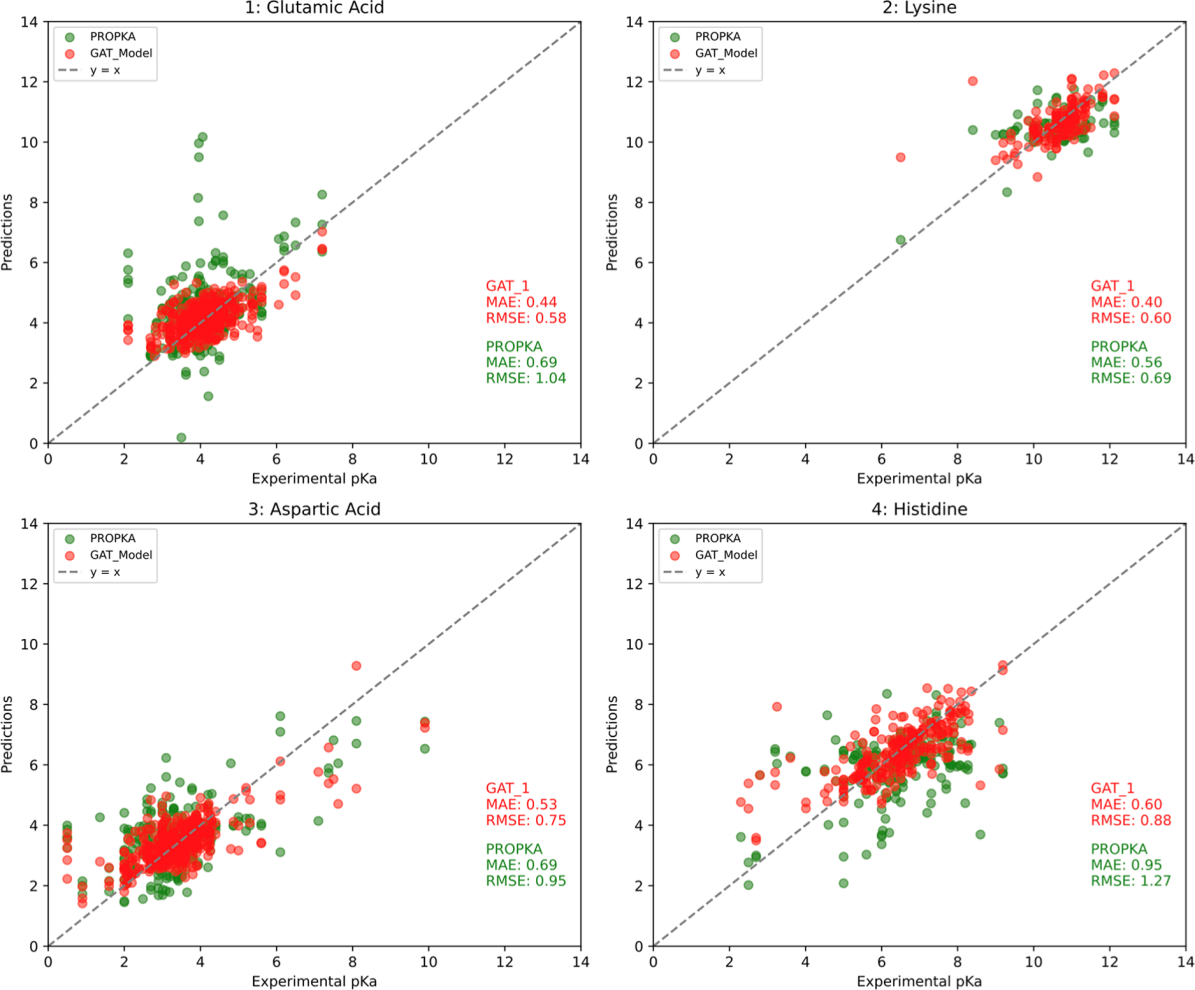
Comparison of 10-fold CV p*K*_a_ predictions from the GAT_1 and PROPKA across four residue types. Red dots represent the predictions made by the GAT_1, while green dots correspond to the PROPKA predictions. The experimental p*K*_a_ values from the PKAD-2 data set are plotted on the *x*-axis, with predicted values on the *y*-axis.

To investigate the systematic deviations, we examined those residues with highly shifted p*K*_a_ predictions from PROPKA3 and noticed that many of those residues have ligands located nearby. Two representative examples are shown in Figure 9. These two examples suggest that a ligand can make the p*K*_a_ values of neighboring residues hard to predict by altering local geometry and electrostatic environment. PROPKA does not explicitly account for the impact caused by those ligands during predictions, but our model can detect the geometric inconsistencies from simulation-derived, physics-based features they learn from, allowing them to better capture these ligand-induced environmental effects.

**Figure 9:**
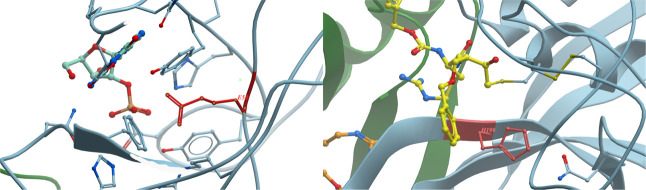
The first case (left) is Glu58 (in red) from 1BVI, which has an experimental p*K*_a_ value of 3.96, but PROPKA predicted an evaluated value of 9.96, while the value predicted by GAT is 3.58. The second one (right) is His199 (in red) from 1THE, with p*K*_a_ reported to be 8.6 in experiments. PROPKA predicted its p*K*_a_ value to be 3.69, while a p*K*_a_ value of 5.32 is predicted from GAT.

While Figure 8 highlights the residue-level prediction accuracy of the GAT_1 model compared to PROPKA for visual evidence of its better alignment with experimental values, it is also important to validate these results within a broader benchmarking framework. To that end, we extended the comparison to include all three top-performing GNN models (i.e., GAT_1, GCN_1, and GIN_1) against null model (which uses the reference p*K*_a_ values as predictions for the corresponding residues, Asp: 3.7, Glu: 4.2, His: 6.5, Lys: 10.4)^28,29^ , PROPKA3.5.1, pKAI+^54^ and DeepKa web server^29,33^ for 796 residues with predictions available across all methods, by a rolling MAE and RMSE for four quantiles, and the quantiles were defined based on the magnitude of the experimental p*K*_a_ shifts relative to the reference p*K*_a_ values (|Δp*K*_a__exp|), to achieve a balanced segmentation of the evaluation set based on both data proportion and p*K*_a_ value range:

- Q1: |Δp*K*_a__exp| within [0.0–0.2], 228 residues (28.6%)
- Q2: |Δp*K*_a__exp| within [0.2–0.5], 186 residues (23.4%)
- Q3: |Δp*K*_a__exp| within [0.5–1.0], 183 residues (23.0%)
- Q4: |Δp*K*_a__exp| within [1.0–6.2], 199 residues (25.0%).

As shown in Figure 10, the GAT_1 model achieved the best overall performance, with MAE values of 0.485 and 0.488, and RMSE values of 0.686 and 0.702, respectively, as well as very similar Q4/Q1 RMSE/MAE Ratios. In the Q1 and Q2 group, notably, the GAT model also yields a MAE of 0.768 and RMSE value of 1.058 in the Q4 group, demonstrating its strong predictive power and robustness in the extreme p*K*_a_ shift cases (|Δp*K*_a__exp| > 1). These results highlight that the GAT model serves not only as a reliable predictor for regular ionizable residue p*K*_a_ values but also as a robust tool for estimating the large p*K*_a_ shifts of ionizable groups in proteins, particularly enzyme-active sites.

**Figure 10:**
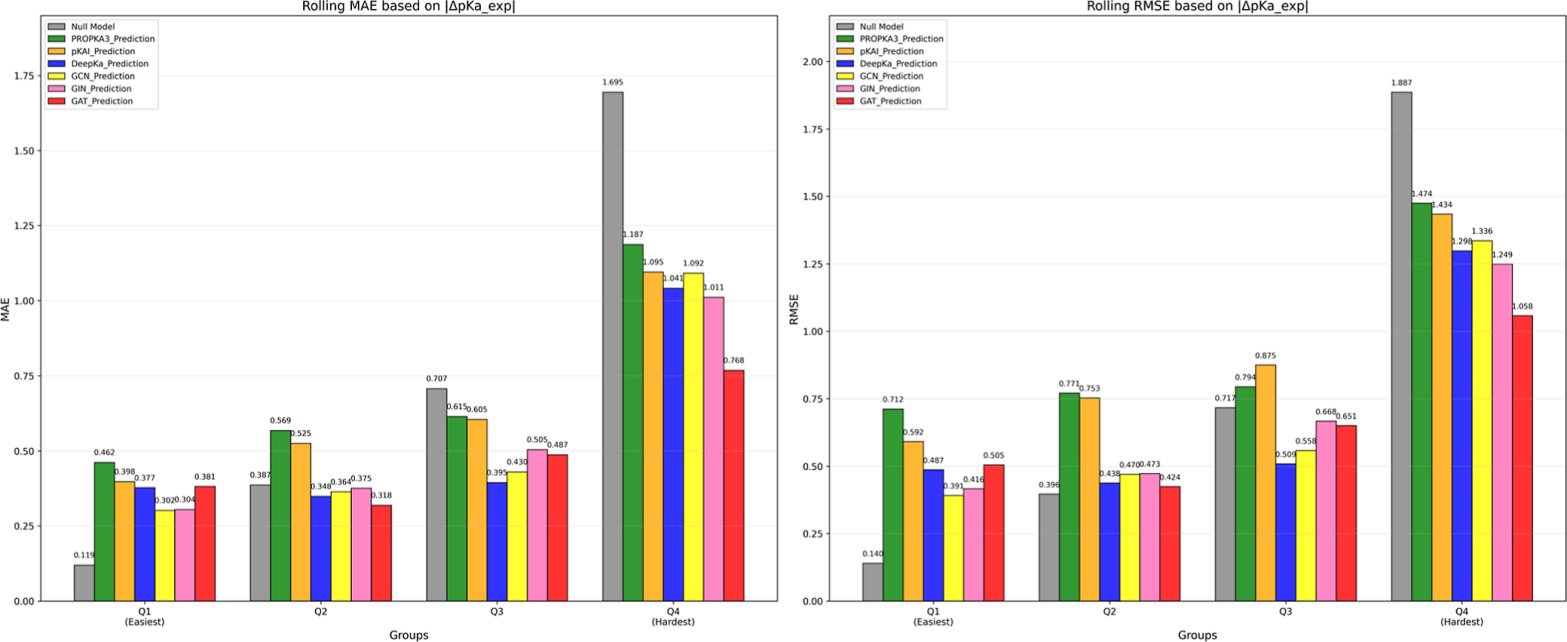
Benchmarking of GNN models against the three predictors using rolling MAE and RMSE.

GAT_1 and DeepKa exhibited a very similar performance across the first three quantiles with GAT_1 showing a much lower MAE and higher RMSE in Q4. The detailed RMSE and MAE for each quantile together with the Q4/Q1 ratio for each predictor are reported in Tables S1 and S2.

The MAE and RMSE values across four residues, based on the benchmark results, are reported in Figure 11. DeepKa-web and GAT achieved comparable performances for His and Glu. While GAT_1 outperformed DeepKa-web for Asp predictions, it had higher MAE and RMSE values for Lys. The detailed RMSE and MAE for each residue type are reported in Tables S3 and S4.

**Figure 11:**
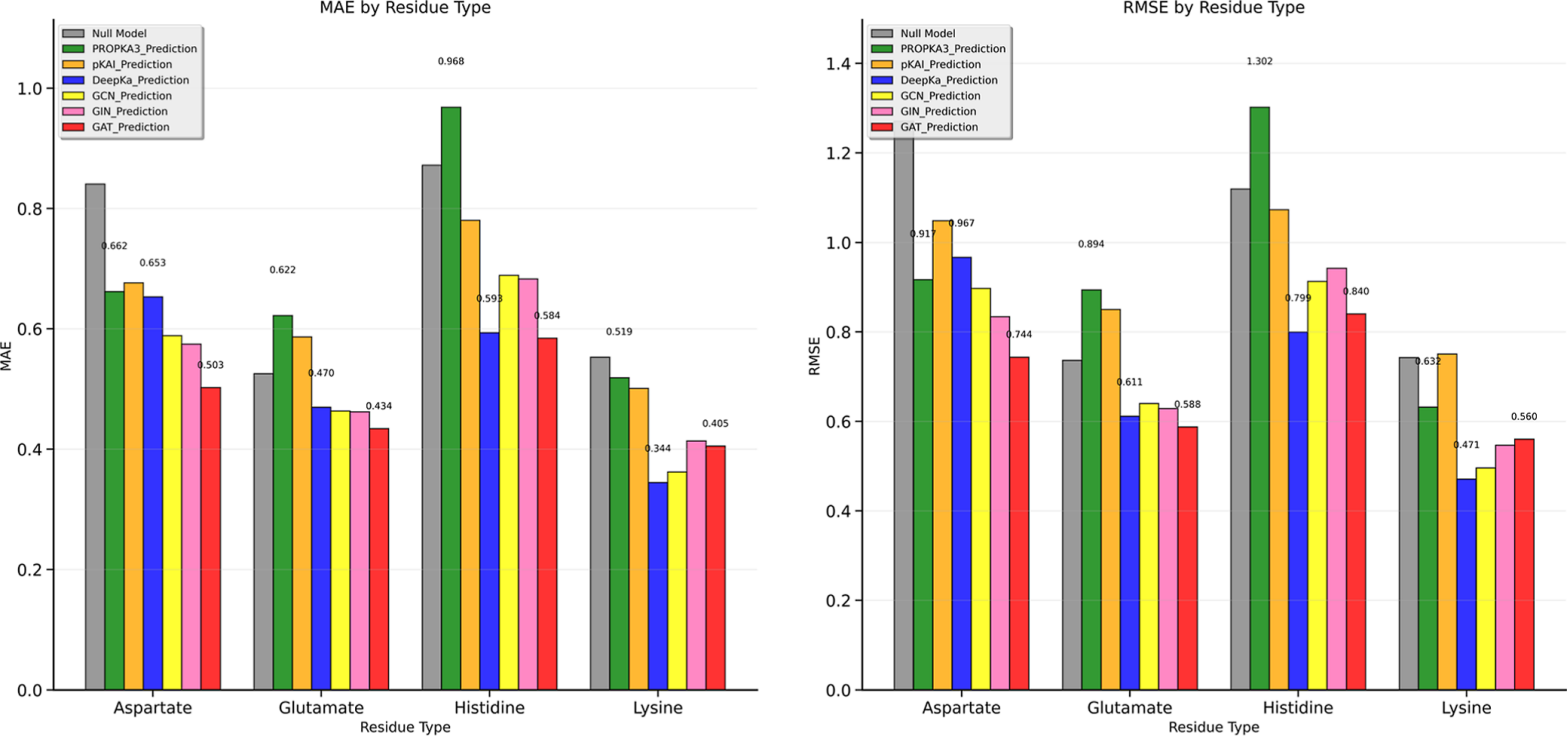
Benchmarking of GNN models against the three predictors, showing a side-by-side MAE and RMSE values’ comparison. The MAE and RMSE values of PROPKA3, DeepKa-web, and GAT_1 are labeled for each residue type.

Since majority of the p*K*_a_ values in the PKAD-2 data set are around reference p*K*_a_ values, metrics like MAE or RMSE alone are not sufficient for model performance evaluation. Therefore, we also calculated correlation coefficient (*R*) shifts of predicted versus experimental p*K*_a_ shifts based on the benchmarking results, reported in Figure S5. Among all models, the GAT_1 model achieved the highest correlation coefficient (*R* = 0.718), and the GIN_1 and GCN_1 models exhibited similar performance, with *R* values of 0.645 and 0.631, respectively, approximately 10% lower than those of GAT, yet about a 5% higher than that of DeepKa-web (*R* = 0.602). In contrast, PROPKA3 and pKAI exhibited the lowest correlation coefficients, with *R* values around 0.5.

To further validate the robustness and generalizability of the models, an independent data set based on PKAD-R was constructed. The residues appear in PKAD-R but are not included in the 1167 residues training set were selected, and the entries corresponding to Cys, Tyr, and C/N-terminal residues were excluded (as discussed before). In addition, mutant proteins lacking complete PDB structures, i.e., mutant proteins with internal deletions, were removed, as complete protein structures were required for simulation process. The resulting data set comprises 83 new protein residues with p*K*_a_ values, mostly from mutant proteins (65 out of 83 residues are from mutant proteins), and the time spent on the energy-minimization step for each protein is reported in Table S6.

Subsequently, the predictions obtained from the ten GAT_1 models (via 10-fold CV) were averaged and benchmarked against those from PROPKA3, DeepKa-Web, and pKAI+. A similar four quantile rolling MAE and RMSE analysis was then performed as shown in Figure 12, and the ranges and sample distributions for each quantile are listed as below:

- Q1: |Δp*K*_a__exp| within [0.0–0.24], 21 residues (25.3%)
- Q2: |Δp*K*_a__exp| within [0.24–0.4], 23 residues (27.7%)
- Q3: |Δp*K*_a__exp| within [0.4–0.9], 18 residues (21.7%)
- Q4: |Δp*K*_a__exp| within [0.9–5.0], 21 residues (25.3%)

**Figure 12:**
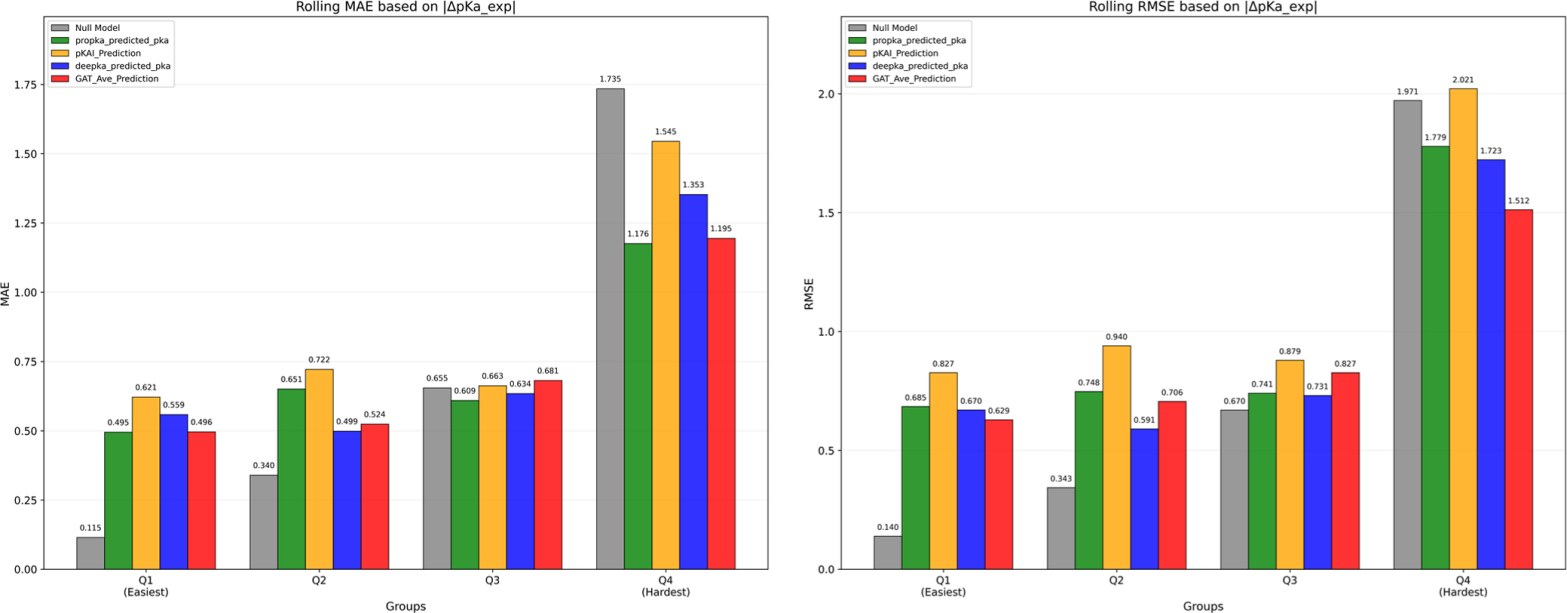
Benchmarking of the GAT_1 model against the three predictors for the predictions from the new mutant data set, with the MAE and RMSE values of PROPKA3, DeepKa-web, and GAT_1 labeled.

The GAT_1 models achieved MAE and RMSE values at 0.72 and 0.98, respectively, and the detailed results for each predictor are summarized in Table 2, with 95% CI provided given the small size of the test set.

**Table 2:** Overall MAE and RMSE of the New Dataset Benchmarking across Four Predictors, with 95% CI Reported

models	samples	MAE	RMSE
PROPKA3	83	0.735 ± 0.166	1.092 ± 0.383
DeepKa-Web	83	0.759 ± 0.145	1.037 ± 0.237
GAT_1	83	0.720 ± 0.139	0.982 ± 0.219
pKAI+	83	0.891 ± 0.188	1.272 ± 0.280

### 3.5 Feature Importance Analysis of the Best-Performing Models

While the sections above evaluate and benchmark the developed p*K*_a_ model performance, it is necessary to further investigate the impact of the 26 features included in the model on the predicted p*K*_a_ values. This information may reveal the physical mechanisms behind the ionizable protein residue p*K*_a_ values determination captured in the developed model, i.e., the influence of local geometric arrangements and the complexity of the surrounding microenvironments on the residue p*K*_a_. To address this end, we employed GNNExplainer,^52^ which has been successfully applied to molecular property prediction task.^53^ In particular, the explanation process in GNNExplainer was implemented by maximizing the mutual information between the model’s predictions *Y* and the learned subgraphs and features (*G*_*S*_,*F*), as formulated in eq 8

8 $$\max_{G_{S},F}MI\;\left(Y,\left(G_{S},F\right)\right)=H\left(Y\right)-H\left(Y|G=G_{S},X=X_{S}^{F}\right)$$

where *G*_*S*_ denotes the explanatory subgraph (including a subset nodes and edges), *F* is a learnable feature mask, and *X*_*S*_^*F*^ represents the masked node features determined by *F*. The goal of this objective function is to maximize the mutual information MI by minimizing the conditional entropy *H*(*Y*|*G* = *G*_*S*_,*X* = *X*_*S*_^*F*^), which enables the framework to identify the substructures and features that are most critical to the model’s prediction on p*K*_a_, thereby offering interpretable insights into the underlying feature importances learned by the developed p*K*_a_ models.

All three best-performing models (i.e., GCN_1, GIN_1, and GAT_1) underwent the same explanation process using GNNExplainer to calculate the feature importance scores, as presented in Figure 13. Overall, the models exhibited highly consistent patterns in the calculated feature important profiles. Among all the features, the number of oxygen atoms within the radii of the corresponding atoms appeared as the most influential feature across all three models, followed by C atom counts. In contrast, the number of nitrogen atoms contributed less to the predictions, and the number of S atoms is even less influential to the predictions.

**Figure 13:**
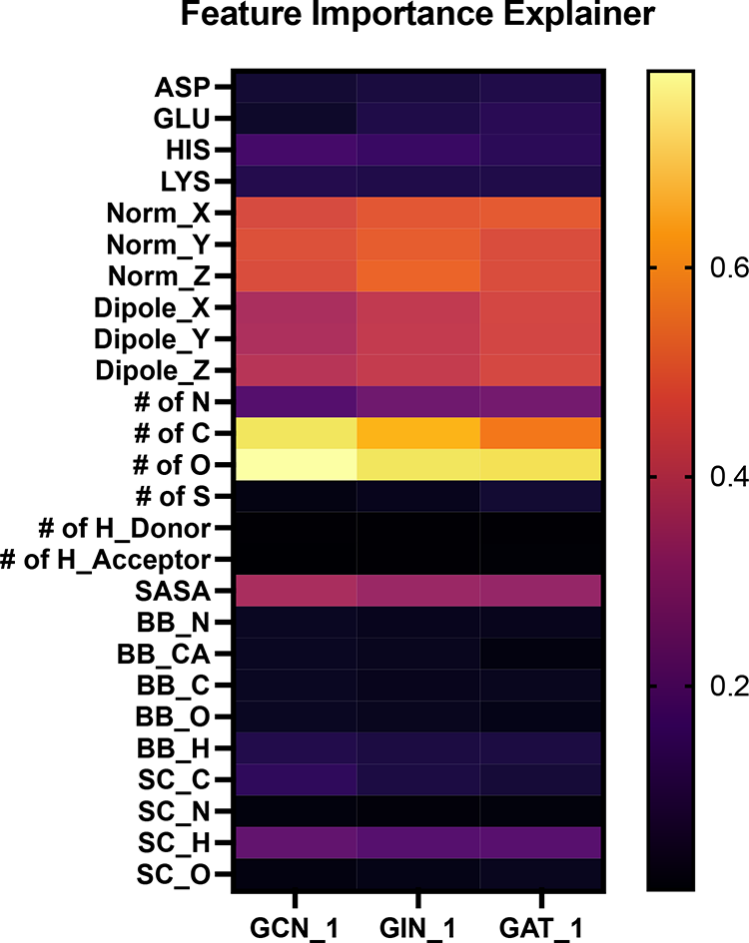
Importance scores of the 26 atomic features across all three best-performing models identified from the study. The lighter the color is, the more important the corresponding feature is.

These results indicate that the models consistently prioritized features based on the physical and chemical principles while making p*K*_a_ predictions. Especially the high importance scores attributed to atomic composition around the atoms, based on previous studies,^55,56^ the electrostatic interactions around ionizable groups, as well as the exposure to the bulk solvent and desolvation, are the dominate contributors to large p*K*_a_ shifts of ionizable residues. These mechanistic insights are consistent with our feature importance analysis results.

Both O and C atoms serve as effective indicators of the complexity of the local environment and the burial level of residues. The number of O atoms is a direct indicator of the local electrostatic interactions, as backbone and side chain carbonyl O can act as strong H-bond acceptors and are major contributors to the local electrostatic field. What is more, negatively charged side chain carboxylates also contribute to the local electrical environment. Second, the number of neighboring carbon atoms reflects the degree of local packing and hydrophobicity. A higher carbon count indicates a more crowded, less solvent-exposed environment and is therefore associated with a stronger desolvation effect. Because carbon atoms do not form strong hydrogen bonds with water and typically carry weak partial charges and because most hydrophobic residues (e.g., Leu, Phe, and Tyr) are carbon-rich, the carbon count also serves as an effective descriptor of the local hydrophobic environment. In contrast, no simple or direct relationship can be established between the nitrogen atom count and the dominant physical determinants of p*K*_a_ shifts. Nitrogen counts are a heterogeneous descriptor: identical nitrogen counts can correspond to markedly different chemical environments. For example, lysine and arginine contain positively charged, highly hydrophilic side chain nitrogens, whereas the nitrogen atom in tryptophan is embedded within a largely hydrophobic aromatic ring. Because these chemically distinct contexts are collapsed into a single scalar feature, the model cannot learn a clear or consistent relationship between nitrogen atom count and p*K*_a_ values and therefore assigns it relatively low importance. Similarly, sulfur atoms are rare in proteins, occurring only in cysteine and methionine, so their local counts exhibit minimal variation across the data set and consequently contribute only weakly to p*K*_a_ prediction.

In addition to the atomic composition of the local environment, two other feature groups also possessed high importance scores: the normalized atomic coordinates within the local frame (i.e., Norm_*X*, Norm_*Y*, and Norm_*Z* in Figure 11) and the atomic dipole moments (i.e., Dipole_*X*, Dipole_*Y*, and Dipole_*Z*). The normalized *X*, *Y*, and *Z* coordinates displayed nearly identical average importance scores across three models. Similarly, the dipole moment vector components in *X*, *Y*, and *Z* directions exhibited consistent importance scores. Following these three sets of high-importance features, the SASA (i.e., SASA) also exhibits a moderate importance.

## 4 Discussion and Limitation

The determination of p*K*_a_ values for protein ionizable residues plays a fundamental role in understanding the functional mechanisms of many proteins and is therefore critical in the early stages of drug discovery, particularly in the identification of target residues. However, despite decades of effort from both experimental and computational approaches, accurately predicting these p*K*_a_ values remains a significant challenge. This difficulty arises from the complicated underlying chemical and physical principles that govern ionization of the residues as well as the lack of accurate representations of both those residues and their residing local environments.

To address these challenges, we developed a graph-neural-network-based framework that integrates induced dipole moments derived from the AMOEBA polarizable FF, which better captures local atomic electrostatics than traditional fixed-point charge models. Unlike conventional FFs, AMOEBA incorporates electronic polarization effects, offering a more realistic depiction of how neighboring atoms influence the local charge distribution, which is an essential factor in determining the ionization potential. In addition to the induced dipole moments, other atomic-level features were extracted from high-throughput MD simulations, including normalized atomic coordinates and descriptors of local environmental complexity. Together, these features were used to construct a physically informed, self-consistent data set that integrates experimentally determined p*K*_a_ values of protein ionizable residues from the PKAD-2 database.

By combining experimentally determined p*K*_a_ values with a novel graph-based representation of each residue’s local environment, the resulting data set served as a robust foundation for training and evaluating three GNN architectures, i.e., GCN, GIN and GAT, in this study. The best-performing models from each architecture were benchmarked against the most widely adopted empirical model PROPKA 3.5.1 as well as the some recently ML-based predictors. The results demonstrated that our GNN models achieved the state-of-the-art, with MAE values around 0.5 p*K*_a_ units, which is within the range of experimental error. Among them, the multi head attention-based GAT_1 model consistently exhibited strong performances and demonstrated good generalizability for residues with large p*K*_a_ value shifts (>1 p*K*_a_ unit) and achieved correlation coefficient *R* of 0.718. Moreover, feature importance analysis revealed that the models prioritized chemically interpretable properties, such as the number of oxygen and carbon atoms, spatial arrangement, and dipole vector, thereby offering insights into the underlying mechanisms governing protein ionizable residues p*K*_a_ shifts.

Despite these promising findings, several limitations remain. First, the study focused on only four ionizable amino acids (i.e., Asp, Glu, His, and Lys) due to their sufficient representation in the PKAD-2 data set. This restricts the applicability of the current models to a subset of residues, excluding important but underrepresented types like Cys and Tyr, as well as C- and N- terminal residues. Terminal residues are intrinsically flexible and often unresolved in experimental structures, leading to their absence in the PDB files. Nevertheless, these residues are ionizable and can significantly impact protein function, which limits the applicability of our models in the covalent drug discovery process. Addressing their exclusions requires additional data; however, experimentally determined p*K*_a_ values for these residues are extremely limited. To augment the data set, synthesized CpHMD calculated p*K*_a_ values or artificially generated data, such as p*K*_a_ predictions from AF2, can be incorporated, as demonstrated in similar studies.^28,29,32,57^ In addition, our models were trained exclusively on the PKAD-2 data set, and while experimentally curated, it may introduce data set-specific biases. Broader evaluation across independent data sets or inclusion of more recent p*K*_a_ measurements would be necessary to establish generalizability. Additionally, the models currently operate on static protein structures and do not account for conformational flexibility or dynamic environmental factors, both of which may influence the ionization behavior in vivo.

Future work will aim to expand the training set with a more diverse array of experimentally determined p*K*_a_ values and incorporate other ionizable residue types by using artificial data. Incorporating ensemble or time-averaged structural features from MD could also enhance the models’ ability to capture context-dependent ionization effects. Overall, our findings represent a promising step toward an accurate, interpretable, and generalizable prediction of protein residue p*K*_a_ values using deep graph learning.

## 5 Conclusions

Accurately predicting the p*K*_a_ values of protein ionizable residues is crucial for understanding biological mechanisms such as enzyme activity, protein–ligand interactions, and pH-dependent conformational dynamics. Despite decades of effort, current methods often fall short due to limitations in how they model the local physicochemical environment of residues. In this work, we introduced a new GNN framework to address these challenges by integrating structural features and induced dipole moments from the AMOEBA-polarizable FF. Our method captures key atomic-level interactions, which were often neglected in traditional models, and encodes them into residue-centered graphs for learning. We evaluated three GNN architectures: GCN, GIN, and GAT, across five data sets derived from the PKAD-2 database. Among the three GNN models, the GAT_1 model achieved the best overall performance, achieving both high prediction accuracy and strong generalizability, even for residues with large p*K*_a_ shifts. Feature importance analysis further confirmed that the models learned meaningful patterns grounded in physicochemical principles identified in this study, such as the number of nearby oxygen and carbon atoms, the spatial positioning of atoms within the local environment, the induced atomic dipole moments, and the SASA, all of which significantly influence the p*K*_a_ values of ionizable residues. These results highlight the power of graph-based learning in modeling complex ionization behaviors. Future work will extend this approach to more residue types, diverse data sets, and dynamic conformations to further enhance generalizability and applicability. The curated dipole moment-enhanced data set and the developed GNN models can serve as valuable resources for the p*K*_a_ prediction community for further model development and validation.

## Supplementary Material



## Data Availability

The models, the curated data sets generated from molecule modeling, and the involved scripts can be found at: https://github.com/Ziyu-S/Graph_pKa.git.

## Notes

Hyperparameter grid search results for the three GNN architecture-based models, training loss and evaluation MAE history for the 10-fold CV GAT_1 model, correlation coefficient *R* of all benchmarked predictors for the WT data set, quantile MAE and RMSE Q4/Q1 ratio of all the benchmarked predictors from WT data set, benchmark results of MAE and RMSE for each residue type from WT data set, and time spent on EM for the proteins in Mutant data set (PDF)
