# Supplementary material for: Graph-Based Deep Learning Models for Predicting pK a Values of Protein-Ionizable Residues via Physically Inspired Feature Engineering
Source: J Chem Inf Model. 2026 Jan 22;66(3):1742–56. doi: 10.1021/acs.jcim.5c01681 (PMC12892328; doi:10.1021/acs.jcim.5c01681)
Supplement: Supplementary file 1 [file ci5c01681_si_001.pdf]

Supplementary Materials:

Graph–Based Deep Learning Models for Predicting pKa Values of Protein Ionizable Residues Via Physically Inspired Feature Engineering

Ziyu Song[1], Ruixuan Wang[2], Xun Jiao[2], Zuyi Huang[1]*

1. Department of Chemical and Biological Engineering, Villanova University, Villanova, PA 19085

2. Department of Electrical and Computer Engineering, Villanova University, Villanova, PA 19085

Correspondence: zuyi.huang@villanova.edu; Tel.: +1–610–519–4848

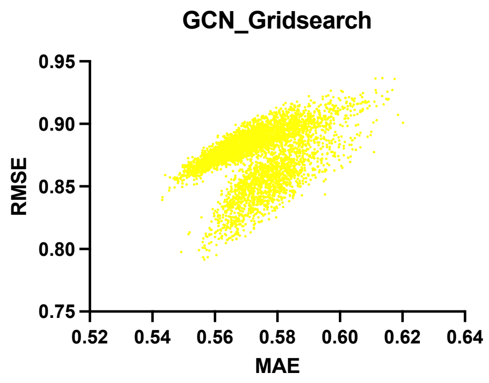

Figure S1: Grid search results showing the MAE and RMSE for all GCN hyperparameter combinations.

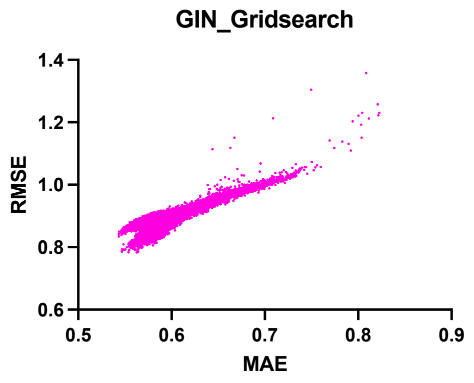

Figure S2: Grid search results showing the MAE and RMSE for all GIN hyperparameter combinations.

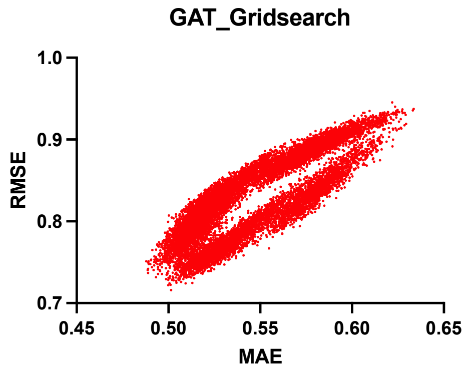

Figure S3: Grid search results showing the MAE and RMSE for all GAT hyperparameter combinations.

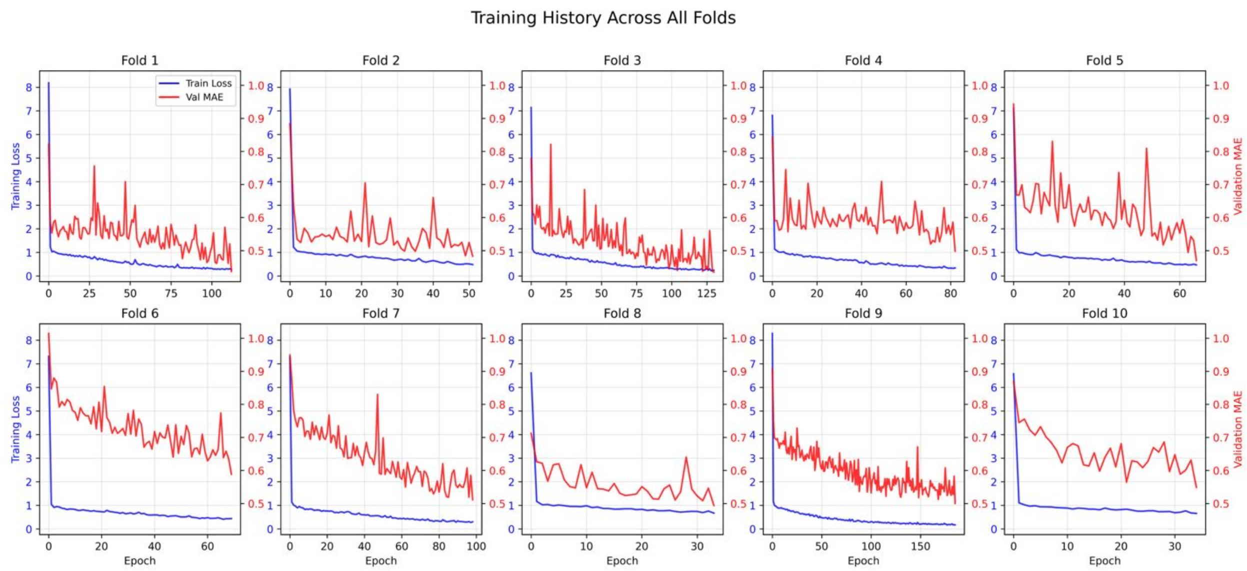

Figure S4: The Training loss and evaluation MAE history for 10-fold CV of the GAT model.

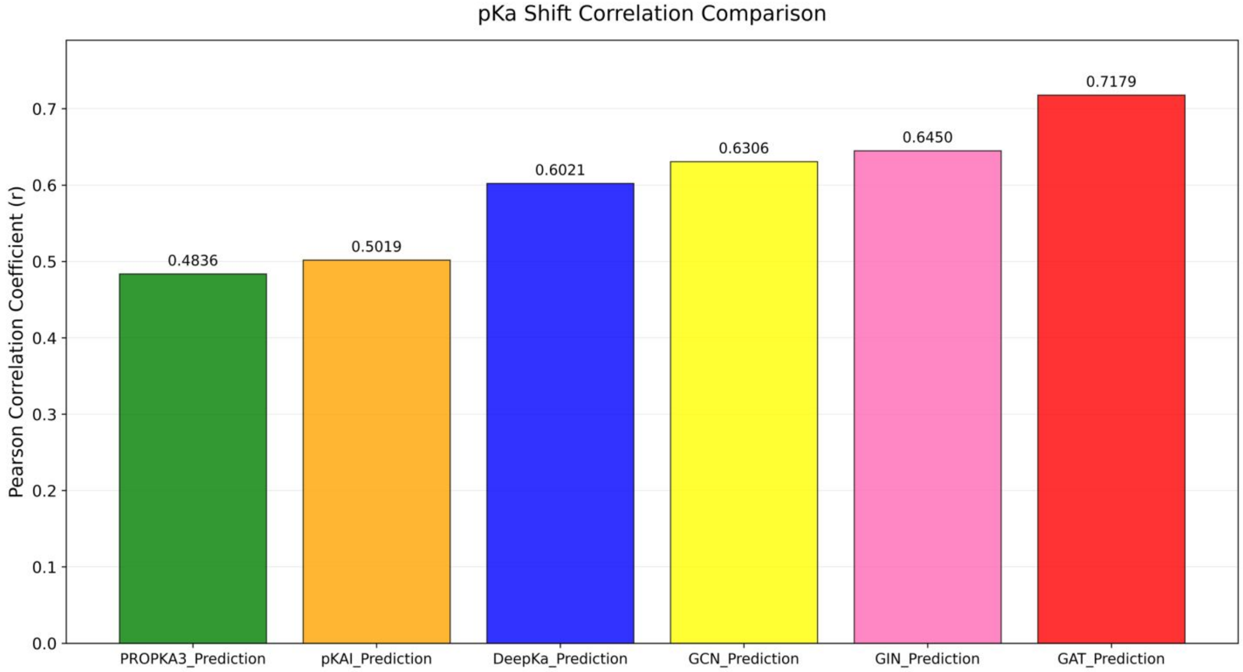

Figure S5: The correlation coefficient R of all benchmarked predictors for WT dataset.

Table S1: The quantile RMSE and Q4:Q1 ratio of all the benchmarked predictors from WT dataset.

Predictor/RMSE	Q1	Q2	Q3	Q4	Overall	Q4:Q1 Ratio
Null Model	0.140	0.396	0.717	1.887	1.025	13.516
PROPKA3	0.712	0.771	0.794	1.474	0.986	2.07
pKAI+	0.592	0.753	0.875	1.434	0.961	2.424
DeepKa	0.487	0.438	0.509	1.298	0.771	2.666
GCN	0.391	0.470	0.558	1.336	0.783	3.413
GIN	0.416	0.473	0.668	1.249	0.771	3.002
GAT	0.505	0.424	0.651	1.058	0.702	2.094

Table S2: The quantile MAE and Q4:Q1 ratio of all the benchmarked predictors from WT dataset.

Predictor/MAE	Q1	Q2	Q3	Q4	Overall	Q4:Q1 Ratio
Null Model	0.119	0.387	0.707	1.695	0.711	14.242
PROPKA3	0.462	0.569	0.615	1.187	0.703	2.57
pKAI+	0.398	0.525	0.605	1.095	0.650	2.753
DeepKa	0.377	0.348	0.395	1.041	0.540	2.762
GCN	0.302	0.364	0.43	1.092	0.543	3.62
GIN	0.304	0.375	0.505	1.011	0.544	3.322
GAT	0.381	0.318	0.487	0.768	0.488	2.015

Table S3: The benchmark results of RMSE for each residue type from WT dataset.

Predictor/RMSE	Asp	Glu	His	Lys
Null Model	1.271	0.737	1.119	0.743
PROPKA3	0.917	0.894	1.302	0.632
pKAI+	1.049	0.85	1.073	0.751
DeepKa	0.967	0.611	0.799	0.471
GCN	0.897	0.64	0.913	0.496
GIN	0.834	0.629	0.942	0.547
GAT	0.744	0.588	0.84	0.56

Table S4: The benchmark results of MAE for each residue type from WT dataset.

Predictor/MAE	Asp	Glu	His	Lys
**Null Model**	0.841	0.526	0.872	0.553
**PROPKA3**	0.662	0.622	0.968	0.519
**pKAI+**	0.676	0.586	0.78	0.501
**DeepKa**	0.653	0.47	0.593	0.344
**GCN**	0.589	0.463	0.689	0.362
**GIN**	0.575	0.462	0.683	0.414
**GAT**	0.503	0.434	0.584	0.405

Table S6: The time spent (in second) on energy minimization for the proteins in Mutant dataset.

File_ID	Time (s)	File_ID	Time (s)
2RVQ.xyz	11157.2777	4EQP.xyz	130.716142
2V8N.xyz	940.382481	5KGU.xyz	129.81401
3DMU.xyz	810.510723	3C1F.xyz	129.215169
2RKS.xyz	551.275685	3EJI.xyz	128.65479
2QDB.xyz	543.483443	3SK5.xyz	126.541483
2RDF.xyz	531.048498	5I6W.xyz	125.448313
1EY7.xyz	496.282165	5E3F.xyz	125.444673
4YIJ.xyz	488.398636	4HMJ.xyz	124.02643
2OXP.xyz	456.803086	3QOL.xyz	123.313918
3D6C.xyz	438.790493	3TP8.xyz	122.01702
3E5S.xyz	409.359014	5J1Z.xyz	121.420139
3D4D.xyz	384.636528	3ERO.xyz	118.558257
7XJD.xyz	344.603861	4BYA.xyz	118.170431
1LE2.xyz	232.958773	3C1E.xyz	117.914151
8UOZ.xyz	223.430358	3ITP.xyz	117.187562
2KIX.xyz	206.932832	5DEH.xyz	115.926825
1QT8.xyz	194.520658	4KY5.xyz	115.772519
1L98.xyz	181.768195	3HZX.xyz	115.714521
1PRW.xyz	173.841429	5KIX.xyz	113.803355
4HMI.xyz	155.017022	4KY6.xyz	113.756979
3QOJ.xyz	149.609219	3BDC.xyz	112.965385
5IIF.xyz	146.841595	3EVQ.xyz	112.765924
1BVC.xyz	144.228945	2OEO.xyz	112.187797
2RBM.xyz	143.096014	6EEG.xyz	110.71141
5JAV.xyz	141.275807	3TP7.xyz	107.660078
5I6Y.xyz	136.990838	3ERQ.xyz	104.914312
3RUZ.xyz	136.398409	5ISR.xyz	104.614957
3H6M.xyz	132.578213	5E1F.xyz	103.834205

(note: Protein 2RVQ is much larger than others.)
